# Reframing Dental Anxiety: Cognitive Behavioral Therapy and Its Role in Phobia Treatment—A Narrative Review

**DOI:** 10.3390/diseases13110377

**Published:** 2025-11-18

**Authors:** Dorina Stan, Dragoș Voicu, Pușica Zainea, Alexandra Toma, Anamaria Ciubară

**Affiliations:** 1 County Emergency Clinical Hospital of Brăila, 810325 Brăila, Romania; 2 Faculty of Medicine and Pharmacy, „Dunărea de Jos” University of Galați, 800201 Galați, Romania; 3 Psychiatric Clinical Hospital „Elisabeta Doamna”, 800179 Galați, Romania

**Keywords:** phobia, dental phobia, cognitive behavioral therapy, dental anxiety, CBT, fear of dentist, dentistry

## Abstract

Dental phobia is a disabling yet underdiagnosed condition that prevents many patients from seeking essential oral healthcare, leading to avoidable pain, disease progression, and reduced quality of life. Cognitive Behavioral Therapy (CBT) is the most widely supported psychological intervention for specific phobias and has demonstrated significant efficacy in reducing dental anxiety and avoidance. This narrative review synthesizes recent evidence on CBT applications for phobia management, with particular emphasis on dental settings. In addition to reviewing established mechanisms of CBT, this paper highlights emerging adjunctive approaches such as virtual reality, eye movement desensitization and reprocessing (EMDR), and hypnosis. Special attention is given to pediatric populations, trauma-exposed individuals, and patients with neurodevelopmental disorders, who are often underrepresented in clinical research. The findings underscore the central role of CBT in addressing dental phobia while identifying gaps in standardized protocols, long-term outcomes, and accessibility across diverse healthcare contexts. Future research should prioritize controlled trials, cultural adaptations, and the integration of psychological training into dental curricula to enhance the translation of evidence into everyday practice.

## 1. Introduction

Phobias are among the most prevalent psychiatric conditions, with lifetime prevalence estimates ranging from 7% to 12% worldwide, and slightly higher rates reported in women and younger adults [1]. They are characterized by intense, persistent fear of specific stimuli or situations, recognized by the individual as disproportionate, and leading to avoidance behaviors that significantly impair daily functioning and health. While phobias have historically been regarded as circumscribed to narrow triggers, their clinical impact is broad: untreated phobias are associated with comorbid depression, generalized anxiety disorder, and substance use, and they frequently interfere with access to medical and dental care [2].

Cognitive Behavioral Therapy (CBT) has been established as the first-line treatment for phobias in most clinical guidelines due to its strong empirical support and durability of outcomes [3]. CBT addresses the cognitive distortions, maladaptive behavioral patterns, and physiological arousal that sustain phobic avoidance, using structured techniques such as cognitive restructuring, graded exposure, and relaxation training [4,5,6]. Unlike pharmacological interventions, which may suppress symptoms temporarily, CBT produces long-term change by modifying the underlying cognitive–emotional mechanisms of fear.

### 1.1. Differentiating Dental Anxiety, Fear, and Phobia

Within this framework, oral health professionals frequently encounter dental anxiety, dental fear, and dental phobia, terms that must be clearly distinguished. Dental anxiety describes a general sense of unease before dental visits, often transient and manageable. Dental fear refers to a stronger emotional reaction to specific triggers (needles, drilling, anesthetic procedures) and is often linked to prior negative experiences. Dental phobia represents the most severe manifestation, meeting diagnostic criteria for specific phobia, marked by persistent, intense fear, avoidance of care, and significant functional impairment [7,8,9]. This differentiation is clinically important because while mild anxiety may be addressed with reassurance and education, phobia requires structured psychological intervention.

### 1.2. Epidemiology and Risk Factors

The prevalence of dental phobia is estimated at 10–20% in adults in industrialized countries, with lower but still clinically relevant rates in some Asian populations [10]. Factors influencing prevalence include age, sex, education, and socioeconomic status. Women consistently report higher rates of dental fear and phobia than men, and childhood adverse dental experiences are strongly predictive of adult dental phobia [11,12]. Genetic vulnerability, temperamental traits such as high neuroticism, and parental modeling of dental avoidance also contribute [13].

### 1.3. Clinical and Systemic Consequences

The consequences extend far beyond the dental office. Avoidance of dental care results in untreated caries, periodontal disease, and tooth loss, which in turn are associated with cardiovascular disease, diabetes, malnutrition, and diminished quality of life [14]. Psychosocially, patients with dental phobia often experience embarrassment, social withdrawal, and heightened depressive or anxious symptoms. On a systemic level, dental phobia contributes to inequalities in healthcare, disproportionately affecting disadvantaged groups with limited access to psychological interventions [15]. Economically, untreated dental anxiety and phobia shift care toward emergency services, which are costlier and less efficient, and they require more invasive procedures when patients finally seek treatment [16].

### 1.4. Comparative Treatment Approaches

A variety of interventions have been proposed. Pharmacological sedation (benzodiazepines, nitrous oxide, general anesthesia) is frequently used for acute symptom management, but its effects are temporary and do not alter the core fear structures. Relaxation techniques and distraction strategies (e.g., music, guided imagery) provide partial relief but lack strong long-term outcomes. In contrast, CBT—with its emphasis on cognitive restructuring and exposure—has demonstrated consistent, durable reductions in avoidance and symptom severity [17]. Recent meta-analytic evidence confirmed that CBT and exposure-based techniques are superior to passive controls and non-specific behavioral methods in reducing dental anxiety and phobia in adults [2].

### 1.5. Emerging Evidence and Innovative Adaptations

Despite robust evidence, treatment effectiveness varies across populations. Vulnerable groups—including children, adolescents, patients with psychiatric comorbidities, and trauma survivors—often require tailored approaches. Case-series data emphasize the importance of interdisciplinary collaboration, where psychological treatment is integrated with dental care [7]. Pediatric systematic reviews indicate that CBT and behavioral management techniques reduce dental fear, though methodological variability remains a limitation [18,19,20].

Technological innovations are expanding access and acceptability. Internet-delivered CBT has been shown to reduce dental anxiety and needle phobia among children and adolescents, offering scalable and cost-effective solutions [21]. Virtual Reality (VR) has emerged as both a distraction tool and an exposure modality. A 2025 scoping review confirmed its ability to reduce pain and anxiety in pediatric dentistry, while randomized controlled trials demonstrated similar benefits in adult populations during invasive procedures [22,23].

Adjunctive psychological approaches are also being explored. Eye Movement Desensitization and Reprocessing (EMDR) may be beneficial for trauma-related dental phobia, while hypnosis has demonstrated moderate effectiveness in reducing procedural anxiety, particularly in patients with high suggestibility [24,25]. Although these methods are not yet as robustly supported as CBT, they may play a complementary role in multidisciplinary care.

### 1.6. Barriers and Gaps

Implementation of CBT for dental phobia faces several barriers. There is a shortage of trained professionals able to deliver CBT in dental contexts, and many dentists report insufficient training in psychological methods. Cultural stigma around psychological therapy also reduces acceptability in some populations. Cost-effectiveness studies are limited, and long-term comparative trials between CBT, VR, and pharmacological sedation are lacking. Moreover, few studies examine outcomes in vulnerable groups such as patients with developmental disorders, older adults, or those with low health literacy [25].

Despite substantial progress, significant research gaps remain. Comparative effectiveness studies directly contrasting CBT with alternative interventions such as pharmacological sedation, hypnosis, or virtual reality are scarce. Evidence regarding pediatric populations and individuals with special healthcare needs is particularly limited, leaving vulnerable groups underserved. Moreover, most available studies report short-term outcomes, with a lack of long-term follow-up data on treatment durability, adherence to dental care, and broader quality-of-life impacts.

### 1.7. Aim of the Review

Taken together, these findings highlight both the progress achieved and the persistent gaps in understanding and applying CBT for dental phobia. The present narrative review with systematic elements aims to consolidate current empirical evidence on CBT in phobia management, examine its specific clinical applications in dental phobia, evaluate innovative delivery formats and adjunctive psychological and technological interventions, and identify research gaps, barriers, and practical implications for integrating CBT into routine dental practice.

## 2. Materials and Methods

This article was conducted as a narrative review with systematic search elements, aiming to synthesize current knowledge regarding Cognitive Behavioral Therapy (CBT) for the treatment of phobias, with particular emphasis on dental phobia. Although not designed as a full systematic review, the study followed a structured and transparent approach to ensure reproducibility and minimize selection bias.

### 2.1. Review Type and Justification

A narrative review methodology was selected due to the interdisciplinary nature of the topic, which spans psychology, psychiatry, behavioral sciences, and dentistry. Evidence in this area is often heterogeneous, with a mix of randomized controlled trials (RCTs), case series, systematic reviews, and theoretical papers. A strict systematic review could have excluded important conceptual or emerging evidence, while a narrative approach allowed for broader inclusion. To strengthen transparency, systematic elements (clear eligibility criteria, structured search, predefined categories for synthesis) were incorporated.

### 2.2. Eligibility Criteria

Inclusion and exclusion criteria were defined before the literature search:

Inclusion criteria:

- Peer-reviewed journal articles, reviews, meta-analyses, RCTs, and case series with transparent methodology.
- Articles published in English.
- Publication period: January 2014–April 2025, with emphasis on the most recent five years (2019–2024).
- Studies addressing phobia treatment with CBT or related psychological interventions.
- Specific focus on dental anxiety, dental phobia, or phobia management in dental contexts.

Exclusion criteria:

- Non-English publications.
- Case reports with insufficient methodological description.
- Editorials, opinion pieces, and conference abstracts without empirical evidence.
- Studies limited exclusively to pharmacological interventions, unless combined with CBT.

### 2.3. Information Sources

Six electronic databases were systematically searched: PubMed, Scopus, PsycINFO, Web of Science, ScienceDirect, and Google Scholar. Searches were performed in April 2025. To enhance completeness, the reference lists of included studies and relevant reviews were also manually screened.

### 2.4. Search Strategy

Search strategies were adapted for each database, combining controlled vocabulary (e.g., MeSH terms in PubMed) with free-text terms. The main concepts used were: phobia, dental phobia or dental phobia, dental anxiety, fear of dentist, cognitive behavioral therapy or CBT, psychological treatment, non-pharmacological intervention, and exposure therapy in dentistry. Boolean operators (AND, OR) were used to refine the results.

The review was developed in line with SANRA (Scale for the Assessment of Narrative Review Articles) recommendations to enhance methodological transparency. An example of the PubMed search string is as follows: (“phobia” OR “dental phobia” OR “specific phobia” OR “dental anxiety” OR “fear of dentist”) AND (“cognitive behavioral therapy” OR “CBT”) AND (“psychological treatment” OR “non-pharmacological intervention” OR “exposure therapy”).

### 2.5. Study Selection

Two independent reviewers screened all records in three stages:

Title screening to eliminate irrelevant papers.

Abstract screening against inclusion and exclusion criteria.

Full-text review for final eligibility.

Any disagreements were resolved by consensus or consultation with a third reviewer. This procedure ensured that the study selection process was transparent and replicable.

**Figure 1:**
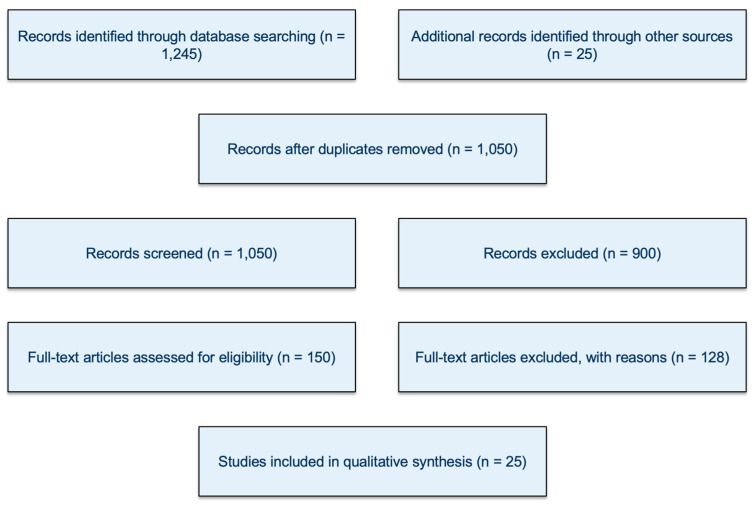
PRISMA Flow Diagram.

Although this review was not designed as a full systematic review, a PRISMA-style flow diagram was included (Figure 1) to increase transparency in reporting the selection process. The diagram illustrates the number of records identified, screened, excluded, and ultimately included in the qualitative synthesis.

### 2.6. Data Extraction

From each eligible study, the following information was extracted using a structured template:

Bibliographic details (authors, year, journal);

Study design and methodological quality;

Sample characteristics (population, age, gender, clinical vs. non-clinical groups),

Type of intervention (CBT protocol, exposure therapy, adjunctive methods such as EMDR, VR-assisted CBT, hypnotherapy);

Outcome measures (e.g., anxiety/phobia scales, treatment adherence, oral health outcomes);

Principal results and conclusions relevant to phobia and dental phobia.

The extraction process was carried out independently by two reviewers and then cross-checked for consistency.

### 2.7. Data Synthesis

Given the heterogeneity of the included studies in terms of design, population, and outcomes, a meta-analysis was not feasible. Instead, a thematic synthesis approach was adopted. Studies were categorized according to three main domains:

Etiological and theoretical models of phobia, with emphasis on dental phobia.

General applications of CBT for specific phobias across clinical settings.

Clinical applications of CBT for dental phobia, including adaptations and interdisciplinary approaches.

This thematic grouping allowed for both conceptual integration and critical comparison of findings, highlighting consistencies, discrepancies, and research gaps.

## 3. Phobias and Their Etiology—Theoretical Approaches

Phobias are complex and multifactorial conditions situated at the intersection of behavioral, cognitive, emotional, and physiological domains. Their classification and etiology reflect broader debates in clinical psychology, from classical conditioning theories to modern biopsychosocial models. Understanding the origins and typologies of phobias, particularly in relation to dental phobia, is essential for evidence-based diagnosis, case formulation, and therapeutic planning.

### 3.1. Classification of Phobias: From DSM to Functional Complexity

The DSM-5 classifies phobias under the umbrella of Anxiety Disorders, distinguishing between the following:

- Specific Phobia, focused on a discrete stimulus (e.g., dentist, injection).
- Social Anxiety Disorder, fear of social scrutiny.
- Agoraphobia, fear of entrapment or helplessness in open/public spaces.

The key diagnostic features of specific phobias include the following:

- Marked and immediate fear or anxiety.
- Active avoidance or intense distress during exposure.
- Recognition that the fear is excessive or unreasonable (in adults).
- Lasting for 6 months or more.

Dental phobia, though formally categorized as a specific phobia, often presents with complex behavioral avoidance patterns, panic-like symptoms, and significant impairment, thus functioning more like a complex phobia in its effects [3]. Patients may go decades without visiting a dentist, leading to social embarrassment, loss of function, and deteriorated physical health [10].

### 3.2. Theoretical Models of Etiology

(a) Psychodynamic Framework: Symbolic Substitution and Inner Conflict

From a psychodynamic perspective, phobias are not irrational per se, but symbolic: they represent internal conflicts displaced onto external stimuli. Freud’s classical case of “Little Hans” (1909) demonstrated how a child’s fear of horses symbolized unconscious fears about castration and paternal punishment.

In dental phobia, such displacement may represent unresolved early traumas involving bodily violation, parental control, or helplessness. A patient with dental phobia may report no conscious dental trauma, yet feel overwhelming dread—possibly rooted in early childhood medical procedures or interpersonal boundary violations [11]. Though less emphasized in empirical studies, psychodynamic insights are valuable in chronic, treatment-resistant cases.

(b) Behavioral Theory: Learning Through Experience and Reinforcement

The behavioral model offers the most robust empirical foundation for phobia development. It posits that fears are acquired via classical conditioning and maintained through operant conditioning:

- A neutral stimulus (e.g., dental drill) becomes associated with an aversive outcome (e.g., pain).
- Subsequent avoidance is negatively reinforced by reduction in anxiety.

Example: A 10-year-old undergoes an extraction without anesthesia. Later, even routine checkups elicit fear. Over time, each cancelation of an appointment relieves distress, reinforcing the cycle.

Vicarious conditioning is equally potent: parents who verbalize fear of dentists or exhibit avoidance may transmit these behaviors intergenerationally [12]. Media and cultural depictions (e.g., the “evil dentist” trope) further exacerbate anticipatory anxiety.

(c) Cognitive Model: Interpretive Biases and Cognitive Distortions

The cognitive model focuses on maladaptive thought patterns:

- Catastrophizing (“I’ll choke and die in the chair”).
- Mind reading (“The dentist thinks I’m disgusting”).
- Fortune-telling (“It will definitely be painful”).

These beliefs, though irrational, are deeply embedded and reinforced by past experiences and societal narratives. Cognitive models also emphasize attentional bias—individuals with dental phobia selectively attend to threat cues like smells, tools, or white coats, amplifying their anxiety response [13].

CBT intervenes by helping patients identify these distortions and test them through graded exposure and cognitive restructuring, leading to symptom reduction and increased behavioral flexibility.

(d) Neurobiological and Evolutionary Perspectives

Recent neuroimaging studies suggest that phobias are linked to hyperactivity in the amygdala and insufficient regulatory input from the prefrontal cortex [8]. This accounts for both the intensity and the resistance to verbal reassurance often seen in phobic reactions.

Evolutionary psychology posits that phobias such as those involving blood or injury (as in dentistry) may reflect evolutionary preparedness—adaptive fears that increased survival in ancestral environments [9]. Thus, dental fear may be biologically predisposed and culturally amplified.

### 3.3. Comorbidity and Societal Implications

Phobias frequently co-occur with the following:

- Major depressive disorder (due to social withdrawal, shame, health deterioration).
- Generalized anxiety disorder (due to chronic worry and hyperarousal).
- Post-traumatic stress disorder (in cases of past dental trauma).
- Substance use disorders (as self-medication for avoidance).

In some cases, dental phobia may co-occur with maladaptive coping behaviors such as alcohol consumption, particularly when depressive symptoms are present [14].

In a recent review, Steenen et al. [2] found that nearly 50% of adults with dental phobia also met criteria for another anxiety or mood disorder. Comorbid conditions complicate treatment, as they require integrative or staged therapeutic approaches.

On a societal level, dental phobia contributes to the following:

- Increased oral health inequality.
- Avoidance of preventive care.
- Higher costs for emergency treatments.
- Reduced quality of life, particularly in vulnerable populations such as children, the elderly, and those with disabilities.

This highlights the need for early screening, interdisciplinary care, and the destigmatization of psychological interventions in dental settings.

## 4. Cognitive Behavioral Therapy in the Treatment of Phobias

Cognitive Behavioral Therapy (CBT) is widely acknowledged as the most effective psychological intervention for the treatment of phobic disorders. Its conceptual foundations lie in the understanding that dysfunctional thought patterns and maladaptive behaviors reinforce and maintain anxiety responses. In the context of specific phobias, including dental phobia, CBT operates on the principle that avoidance of feared stimuli prevents individuals from confronting and restructuring their catastrophic beliefs, thus perpetuating the anxiety cycle [13].

The primary mechanism through which CBT achieves therapeutic change is by helping individuals identify and modify negative automatic thoughts that occur in response to phobic stimuli. These thoughts often include overestimations of danger, underestimations of coping ability, and catastrophic predictions about the outcome of exposure. In dental phobia, for instance, a patient may believe that “the dentist will cause unbearable pain” or “I will panic and lose control during the procedure.” Such thoughts lead to intense emotional distress and behavioral avoidance, which in turn prevent the disconfirmation of irrational beliefs. CBT aims to disrupt this self-reinforcing loop through a structured process of cognitive restructuring and controlled exposure [15].

A typical CBT protocol for phobia treatment begins with psychoeducation, during which the therapist explains the cognitive-behavioral model and how thoughts, emotions, and behaviors interact to sustain anxiety. This is followed by the development of a fear hierarchy—an individualized list of anxiety-provoking situations ordered from least to most distressing. In the case of dental phobia, the hierarchy might include thinking about making a dental appointment, entering the dental office, sitting in the waiting room, hearing the sound of the drill, and finally undergoing an examination or procedure.

Systematic exposure to the items on the fear hierarchy is a central component of CBT. Patients are gradually and repeatedly exposed to feared situations in a controlled manner, starting with those that provoke only mild anxiety. Over time, this process leads to habituation—a natural decrease in emotional reactivity—and new learning that contradicts catastrophic beliefs. When patients remain in the feared situation without escaping, they begin to recognize that their anxiety diminishes naturally and that the anticipated disaster does not occur. This corrective experience plays a crucial role in building self-efficacy and reducing avoidance behavior [12].

Another essential element of CBT is cognitive restructuring. This involves identifying the distorted thinking patterns that contribute to the maintenance of fear and systematically evaluating their accuracy. For example, a patient with dental phobia might work with the therapist to examine the evidence for and against the belief that dental procedures always result in unbearable pain. Through guided discovery and Socratic questioning, patients learn to replace maladaptive cognitions with more realistic and balanced thoughts. This process not only reduces anxiety in the short term but also enhances long-term resilience by reshaping core beliefs about safety, control, and vulnerability [7].

In addition to traditional techniques, modern adaptations of CBT have incorporated emerging technologies such as Virtual Reality Exposure Therapy (VRET). This approach allows patients to confront simulated dental scenarios in a controlled, immersive environment, offering a bridge between imaginal and in vivo exposure. Studies suggest that VRET is particularly effective for individuals who are unwilling or unable to engage in direct exposure initially, and may increase treatment engagement and adherence [7].

Empirical evidence strongly supports the efficacy of CBT for phobia treatment. Meta-analyses report large effect sizes, with improvements often maintained at follow-up assessments. For example, Armfield et al. [16] found that exposure-based CBT significantly outperformed placebo, relaxation training, and waitlist controls in reducing phobic symptoms. More specifically, Steenen et al. [2] conducted a systematic review on dental phobia and concluded that CBT interventions resulted in clinically meaningful reductions in dental anxiety scores, improved treatment compliance, and long-term decreases in avoidance behaviors. These outcomes have consistently proven more sustainable than those achieved through pharmacotherapy, which may alleviate symptoms in the short term but does not address the underlying cognitive and behavioral mechanisms.

When compared to other non-pharmacological interventions such as hypnotherapy or Eye Movement Desensitization and Reprocessing (EMDR), CBT remains the most robust and evidence-based option. Hypnotherapy has shown some utility in reducing procedural anxiety, but the results are inconsistent and often depend heavily on therapist expertise and patient suggestibility [17]. EMDR may be beneficial for patients with trauma-related dental phobia; however, current research does not support its superiority over CBT in standard phobia cases [3].

Despite its high efficacy, CBT is not without limitations. Individual differences such as optimism and the ability to manifest positive affect have been shown to influence psychological treatment outcomes, including CBT for anxiety and phobia disorders [18]. A minority of patients may exhibit limited response due to severe avoidance, comorbid psychiatric conditions, or low treatment motivation. In such cases, integrating CBT with other modalities, such as motivational interviewing or pharmacological support, may enhance outcomes. Furthermore, adaptations of CBT are necessary for specific populations, including children, the elderly, and individuals with intellectual or developmental disabilities. For instance, pediatric protocols often involve behavioral modeling, play-based exposure, and caregiver involvement, while older adults may benefit from simplified cognitive interventions and slower pacing [4,10].

In conclusion, Cognitive Behavioral Therapy remains the best supported intervention for the treatment of phobias, including dental phobia, due to its structured approach, strong empirical foundation, and adaptability across populations. By combining cognitive restructuring with graduated exposure, CBT enables individuals to confront their fears, develop coping strategies, and ultimately regain control over their behavioral and emotional responses to dental care.

## 5. Dental Phobia: Prevalence, Causes and Clinical Manifestations

Dental phobia, also referred to in clinical contexts as dental phobia or dental phobia, is a distinct and debilitating form of specific phobia characterized by excessive, irrational fear related to dental procedures. More severe than general dental anxiety, dental phobia typically results in chronic avoidance of dental care, even in cases of urgent need, and often presents with both physiological arousal and cognitive distortions [2]. Individuals affected by this condition frequently experience tachycardia, nausea, trembling, and panic attacks at the mere anticipation of a dental visit. These responses are often disproportionate to the actual stimuli encountered and are fueled by learned associations and perceived threats to safety and control.

The global prevalence of dental phobia varies, but studies consistently report that between 10 and 20% of adults exhibit clinically significant fear that interferes with dental attendance [1]. Higher rates are commonly observed among women, younger individuals, and those with low socioeconomic status or limited access to dental education [5]. Particularly vulnerable are populations with previous traumatic dental experiences, or those who have witnessed fearful or avoidant behaviors in others—most often parents or caregivers—supporting the role of vicarious learning in the development of this condition [3].

Dental anxiety is not limited to the general population; it has also been identified among individuals with medical training, suggesting that even healthcare knowledge does not necessarily buffer against fear responses in dental settings [26].

The etiology of dental phobia is multifactorial. Traumatic experiences, especially during early dental care encounters, are the most frequently cited causes. Painful procedures performed without adequate explanation or control can leave a lasting impression and contribute to the formation of negative expectations. Alongside direct conditioning, the observation of anxiety in family members or cultural depictions of dentistry as inherently painful can create deep-rooted fear responses. Additional contributing factors include personality traits such as high neuroticism, general health anxiety, and comorbid psychiatric disorders like panic disorder, generalized anxiety disorder, and post-traumatic stress disorder, all of which may amplify or maintain dentophobic symptoms [2,5].

Clinically, dental phobia manifests as a complex interplay of emotional, cognitive, behavioral, and physiological components. Patients often exhibit anticipatory anxiety days or even weeks before a scheduled appointment. During actual exposure to dental environments, symptoms may escalate dramatically, including hyperventilation, fainting, disorientation, and in severe cases, full panic attacks. These symptoms are often compounded by cognitive distortions such as catastrophizing (“I might die in the chair”) and negative self-evaluation (“They’ll judge me for my bad teeth”), which reinforce avoidance and emotional distress [3].

To assess the intensity and nature of dental fear, several psychometric instruments have been developed. Among the most commonly used is the Modified Dental Anxiety Scale (MDAS), a five-item questionnaire that evaluates anxiety related to specific dental scenarios. A total score of 15 or higher is indicative of high anxiety, while scores above 19 suggest the likely presence of dental phobia [17]. Another widely utilized measure is the Dental Anxiety Scale—Revised (DAS-R), which, although slightly less detailed, remains a valid screening tool, particularly in general practice. A more clinically nuanced classification is offered by the Seattle System, which categorizes fear into four dimensions: fear of specific stimuli, distrust of dental professionals, general anxiety regarding dental treatment, and embarrassment or shame over oral health status. This model is particularly useful in tailoring interventions, as it aligns well with CBT approaches that target specific fear types [3].

The repercussions of untreated dental phobia are significant, both at the individual and societal levels. On a personal level, patients with dental phobia are more likely to experience advanced dental decay, periodontal disease, tooth loss, and infections due to prolonged avoidance of preventive care. Notably, these consequences often necessitate more invasive and painful interventions, which in turn exacerbate fear and reinforce avoidance behavior. Psychosocially, individuals may suffer from poor self-esteem, social withdrawal, and impaired quality of life related to speech, nutrition, and interpersonal relationships [5].

At the public health level, dental phobia contributes to systemic burdens, including increased reliance on emergency dental services, higher treatment costs, and unequal access to care. Those who suffer from dental phobia are up to five times more likely to delay dental visits for five years or longer, typically returning only during episodes of acute pain or infection, at which point treatment is more complex and costly [2].

## 6. Clinical Applications of Cognitive Behavioral Therapy (CBT) for Dental Phobia

The application of Cognitive Behavioral Therapy (CBT) to the treatment of dental phobia represents a significant advancement in both psychological and dental practice. Unlike general approaches to dental anxiety that rely solely on reassurance or sedation, CBT offers a structured, evidence-based framework for addressing the underlying cognitive and behavioral mechanisms that maintain fear and avoidance.

One of the defining features of CBT in the context of dental phobia is its customizability to the individual’s fear profile. Based on diagnostic frameworks such as the Seattle System and assessment instruments like the Modified Dental Anxiety Scale (MDAS), therapists and dental professionals can collaboratively identify whether the dominant fear relates to specific stimuli (e.g., needles, pain), loss of control, distrust in dental professionals, or social embarrassment. This precision allows for the selection of targeted cognitive and behavioral strategies that match the patient’s psychological profile [3].

In clinical settings, CBT for dental phobia typically begins with psychoeducation, where patients are introduced to the anxiety model and the rationale behind the therapeutic process. This is particularly important in cases of dental phobia, where individuals often believe that their fear is “irrational” or shameful. By normalizing the fear response and explaining how avoidance behavior reinforces anxiety, therapists help patients shift from self-blame to a more constructive understanding of their symptoms [12].

Following psychoeducation, the therapist works with the patient to construct a fear hierarchy based on individual triggers. For example, a patient might rate the act of calling to schedule a dental appointment as mildly anxiety-inducing, while sitting in the chair or hearing the drill may evoke panic. This hierarchy becomes the foundation for a gradual, step-by-step exposure process. The patient is supported through each stage with cognitive restructuring, where maladaptive thoughts are identified, challenged, and replaced with more realistic alternatives. In cases of dental phobia, thoughts such as “I will suffocate during the procedure” or “The dentist will judge me” are re-evaluated using evidence from prior experiences, therapist feedback, and real-life behavioral testing.

One particularly effective technique in dental settings is in vivo exposure, which involves the patient entering the actual environment of fear under controlled conditions. Collaboration with dental practitioners is crucial here, as dental staff must be trained to allow flexibility, offer verbal support, and avoid unexpected or abrupt interventions. Exposure sessions may begin with non-invasive activities, such as visiting the clinic and sitting in the waiting area, and progressively build toward tolerance of full treatment procedures. Evidence suggests that when this method is combined with relaxation training—such as deep breathing, progressive muscle relaxation, or mindfulness—it can significantly reduce anxiety and enhance emotional regulation [7].

In more technologically advanced clinical environments, Virtual Reality Exposure Therapy (VRET) is also being integrated into CBT protocols. VRET allows patients to interact with immersive dental simulations, including sounds, sights, and even the sensation of reclining in a chair. This method has proven particularly beneficial for patients with high avoidance levels or those who refuse real-life exposure in early stages of treatment. VRET has demonstrated comparable efficacy to traditional exposure therapy while improving engagement and patient acceptability [2].

Importantly, CBT for dental phobia often requires interdisciplinary collaboration. Dentists play an essential role not only in identifying anxious patients but also in facilitating therapeutic processes. Programs that integrate psychological screening and brief interventions into dental practices—such as pre-treatment CBT sessions or joint appointments with a therapist and dentist—have been shown to improve treatment adherence and reduce procedure-related distress [4].

A growing body of research supports the long-term efficacy of CBT for dental phobia. Unlike sedation or pharmacological approaches, which offer temporary relief, CBT provides patients with internal coping mechanisms that generalize across situations. Follow-up studies indicate that patients treated with CBT are more likely to maintain regular dental attendance, show improved oral hygiene, and experience a greater sense of autonomy and control over their health behaviors [3,16].

However, several barriers remain. Some patients may be reluctant to engage in therapy due to stigma, lack of awareness, or logistical constraints. Others may present with comorbid conditions such as generalized anxiety or depression, which require broader therapeutic focus. In such cases, adapted or blended approaches—including motivational interviewing, brief CBT formats, or digital CBT platforms—can offer more accessible alternatives. Additionally, therapists must be sensitive to cultural differences in emotional expression, perceptions of health professionals, and expectations regarding pain and control, adapting techniques accordingly [5].

In conclusion, the clinical application of CBT for dental phobia marks a shift toward integrated, patient-centered care in dentistry. By addressing the psychological roots of dental fear through structured interventions, CBT not only reduces avoidance and distress but also restores the possibility of regular, preventive dental care. As more dental clinics incorporate psychological services and more therapists receive training in dental-specific CBT, the pathway to treatment becomes more accessible, efficient, and humane for individuals with this often-hidden yet profoundly impactful condition.

## 7. Discussion

The evidence synthesized in this review underscores the central role of Cognitive Behavioral Therapy (CBT) in the management of dental phobia, while also highlighting the rapid evolution of adjunctive approaches and the persistent challenges that limit widespread clinical implementation. Beyond confirming the efficacy of CBT, the findings suggest that the next phase of progress requires a multidimensional strategy—integrating psychological science, technological innovation, and health systems reform.

### 7.1. CBT as a Mechanism-Based Intervention

CBT remains the most rigorously studied psychological intervention for specific phobias, including dental phobia. Its superiority over passive and non-specific behavioral strategies has been established across multiple meta-analyses [2]. Unlike sedation or pharmacological interventions, which provide short-lived relief, CBT modifies the underlying mechanisms of fear learning and avoidance. Cognitive restructuring targets catastrophic misinterpretations (e.g., “dental treatment will cause unbearable pain”), while graded exposure facilitates extinction learning, recalibrating neural circuits linking the amygdala, hippocampus, and prefrontal cortex [5].

Neuroimaging studies further support CBT’s mechanistic plausibility, showing reduced amygdala reactivity, enhanced prefrontal modulation, and strengthened functional connectivity in fear-regulation circuits. These findings align with cognitive models that emphasize the role of maladaptive threat appraisals, attentional biases, and avoidance behavior in maintaining phobic responses. In this sense, CBT does not merely reduce symptoms but addresses the very architecture of fear learning.

### 7.2. Comparison with Alternative Treatments

Pharmacological approaches—including benzodiazepines, nitrous oxide sedation, and general anesthesia—remain common in dental practice. These interventions can ensure short-term procedural compliance but fail to address long-term phobia maintenance. Furthermore, risks of dependency, side effects, and increased healthcare costs make them less sustainable solutions.

Educational and relaxation-based strategies (e.g., guided imagery, deep breathing, music therapy) may reduce anticipatory anxiety but show limited durability when compared to CBT [17]. Even advanced pharmacological protocols, such as conscious sedation with midazolam, are not curative and must be repeated for each procedure, highlighting the need for psychological methods that produce lasting change.

Emerging psychological modalities such as Acceptance and Commitment Therapy (ACT) and mindfulness-based programs emphasize acceptance of distress rather than elimination of fear. While promising in pilot trials, these approaches lack the robust empirical base of CBT and have not yet demonstrated superiority in dental phobia contexts.

### 7.3. Pediatric and Developmental Considerations

Dental anxiety often originates in childhood, sometimes after a single traumatic dental event. Without early intervention, these fears crystallize into persistent phobia. Recent systematic reviews confirm that behavioral interventions and CBT techniques are effective in pediatric settings [19,20]. Importantly, internet-delivered CBT (iCBT) offers scalable solutions for younger populations, combining accessibility with efficacy [18].

Integration of caregivers into CBT protocols and the implementation of school-based dental anxiety prevention programs represent underexplored but potentially powerful strategies. Early, preventive interventions could interrupt the trajectory from mild childhood anxiety to chronic adult phobia.

Children with neurodevelopmental disorders (e.g., autism spectrum disorder, intellectual disability) represent a particularly underserved group. Standard CBT requires adaptation—simplified cognitive techniques, slower pacing, visual aids, and heavy reliance on behavioral strategies. Collaboration between pediatric dentists, child psychologists, and caregivers is essential but rarely studied systematically. Future research must prioritize inclusive designs that test interventions in these vulnerable groups.

### 7.4. Trauma and Vulnerable Populations

Trauma-exposed individuals frequently present with severe dental phobia, often linked to medical or dental maltreatment, sexual abuse, or other adverse life experiences. Interdisciplinary treatment models, where CBT is delivered in coordination with dental practitioners, have shown promising outcomes [7].

Trauma-informed dental care involves predictable routines, transparent communication, gradual exposure, and sensitivity to triggers. Integrating trauma screening into dental intake assessments may help identify at-risk patients early and ensure timely referral. This subgroup exemplifies the broader principle that effective phobia management requires personalization and contextual awareness.

### 7.5. Technological Innovations: VR and Digital Platforms

The rapid growth of Virtual Reality (VR) interventions has transformed the landscape of dental anxiety management. VR can function both as immersive distraction and as simulated exposure. Scoping reviews confirm reductions in pain and anxiety among children undergoing routine dental procedures [21]. Randomized controlled trials further demonstrate benefits in adults undergoing invasive extractions [22].

Recent evidence also suggests that VR may not be equally effective across all populations and contexts. Pediatric patients and individuals with special healthcare needs appear to benefit most, as immersive distraction can reduce both procedural anxiety and pain perception during repeated or invasive treatments. VR-based audiovisual distraction tools, as highlighted by Shetty et al. [27], have been particularly effective in multi-session pediatric dentistry, where traditional chairside methods often fail to maintain engagement. In contrast, for adults undergoing complex procedures, VR may have limited impact when compared to graded in vivo exposure, and its benefits may diminish over time if not integrated within structured CBT protocols. These findings indicate that VR should be viewed not as a universal solution, but as a valuable adjunct tailored to specific patient groups and clinical scenarios.

However, most studies evaluate short-term outcomes. Long-term effectiveness, cost-effectiveness, and optimal VR content design remain unclear. Comparative studies are required to establish whether VR-enhanced exposure confers additional benefit beyond standard in vivo techniques, or whether it primarily functions as a preparatory tool.

Digital delivery of CBT extends beyond VR. Internet-based platforms and mobile applications provide structured CBT modules, sometimes supplemented with therapist guidance. These approaches expand reach, reduce costs, and normalize help-seeking behavior. Artificial intelligence–driven personalization may further refine treatment, adjusting exposure hierarchies and cognitive tasks in real time. Biofeedback integration could allow interventions to adapt dynamically to physiological arousal. Yet, regulatory, ethical, and privacy issues remain barriers to clinical deployment.

### 7.6. Adjunctive Psychological Interventions

EMDR and hypnosis illustrate the growing diversification of psychological approaches. EMDR may be especially relevant for patients with trauma histories, as bilateral stimulation facilitates reconsolidation of traumatic memories [23]. Hypnosis, supported by meta-analytic evidence in dental and broader medical contexts [24,25], offers potential transdiagnostic benefits.

Despite promising evidence, barriers to adoption include variable training standards, limited practitioner availability, and skepticism within the dental community. These approaches may serve best as adjuncts to CBT or as alternatives in cases where CBT is inaccessible, unacceptable, or ineffective.

To provide a concise synthesis and facilitate comparison across approaches, Table 1 summarizes the main interventions for dental phobia, highlighting their target populations, evidence base, clinical outcomes, and key limitations.

As illustrated in Table 1, while multiple interventions show potential benefits, CBT consistently demonstrates the strongest evidence base and durability of outcomes, supporting its role as the cornerstone of dental phobia management.

Sociocultural and Economic Barriers

Cultural differences shape both the prevalence of dental phobia and attitudes toward psychological treatment. In some societies, stigma surrounding mental healthcare reduces acceptability of CBT referrals.

Economic disparities further exacerbate inequities: while high-income countries increasingly explore VR and iCBT, low- and middle-income countries struggle to implement even basic CBT services.

Healthcare system factors also play a crucial role. Most dental practices lack embedded psychological support, and referral pathways to mental health professionals are often fragmented. Reimbursement structures rarely cover psychological interventions in dental settings, creating financial disincentives.

Cost-effectiveness research is urgently needed to demonstrate that upfront investment in CBT and related interventions reduces long-term expenditures by decreasing emergency visits and invasive procedures.

### 7.7. Research Gaps and Future Directions

Despite a robust literature base, significant gaps persist. Large, multicenter RCTs comparing CBT with VR, EMDR, hypnosis, and pharmacological sedation are lacking. Cost-effectiveness and implementation studies are scarce. Vulnerable populations remain underrepresented, and outcomes such as long-term adherence to dental care, quality of life, and systemic health impacts are seldom reported.

Advances in neuroimaging and psychophysiology offer opportunities to link CBT outcomes with biomarkers of fear regulation, but such translational research is still in its infancy. Future research should adopt implementation science frameworks, focusing not only on efficacy but also on feasibility, acceptability, and sustainability in real-world practice. Hybrid effectiveness–implementation trials could provide critical insights into how CBT and adjunctive interventions can be scaled across diverse healthcare systems. Overall, the existing evidence must be interpreted with caution. The reviewed studies display considerable variability in treatment protocols, outcome measures, and follow-up duration. Many trials included small and heterogeneous samples, which limits statistical power and generalizability. In addition, publication bias cannot be ruled out, as most studies report favorable results while negative or null findings remain underrepresented. These factors highlight the need for more consistent, large-scale, and methodologically transparent research.

### 7.8. Limitations

This review has several limitations that should be acknowledged. First, as a narrative review with systematic elements, the selection of studies may have been influenced by publication bias and the availability of English-language sources. Second, the heterogeneity of study designs, populations, and outcome measures precluded meta-analytic synthesis, limiting the ability to quantify effect sizes. Third, most included studies reported short-term outcomes, making it difficult to draw firm conclusions about the long-term durability of interventions. Finally, while we attempted to cover a broad interdisciplinary scope, some emerging approaches may not have been fully captured. These limitations should be taken into account when interpreting the findings and underscore the need for more rigorous, comparative, and longitudinal research.

Moreover, several methodological sources of bias should be acknowledged. The included studies showed variability in design quality, sample size, and reporting standards, which may have influenced outcome interpretation. The absence of dentophobia-specific randomized controlled trials (RCTs) further limits the strength of causal inferences. Publication bias is also likely, as most available studies report positive findings. These factors emphasize that current evidence, while encouraging, must be interpreted with caution and validated through larger, better-controlled studies.

Formal risk of bias assessment and quality appraisal were not performed due to the narrative design. However, systematic elements were applied to enhance transparency, including predefined eligibility criteria, independent dual screening, structured data extraction, and the inclusion of a PRISMA-style flow diagram.

### 7.9. Practical Implications and Recommendations

The evidence summarized in this review has several direct implications for clinical practice and healthcare systems. First, routine dental intake should incorporate brief anxiety and phobia screening tools, enabling early identification of patients at risk of treatment avoidance. Second, dentists should be trained in basic behavioral management techniques, such as communication strategies, graded exposure, and integration of relaxation methods, while more severe cases should be referred to mental health professionals trained in CBT. Third, technological adjuncts such as VR and internet-delivered CBT (iCBT) can expand accessibility, particularly for pediatric patients and individuals in underserved or remote areas. Fourth, health policy reforms are needed to integrate psychological services within dental care, including reimbursement pathways and interdisciplinary collaboration models. Finally, greater investment in implementation research will be critical to translate evidence-based interventions into everyday dental practice.

### 7.10. Strategic Implications

Taken together, the evidence emphasizes that CBT is the cornerstone of treatment for dental phobia, but it must be integrated into a broader, multidisciplinary framework. For clinicians, this means combining psychological and dental expertise to deliver patient-centered care. For researchers, the priority lies in rigorous trials that include vulnerable populations, employ long-term follow-up, and assess cost-effectiveness. For policy makers, the challenge is to create funding and training infrastructures that enable widespread adoption of CBT and its digital or technological extensions.

## 8. Conclusions and Recommendations

Cognitive Behavioral Therapy (CBT) remains the most consistently supported psychological intervention for managing dental phobia, demonstrating strong evidence for reducing fear, avoidance, and treatment non-adherence. However, the evidence base is still limited by the small number of dentophobia-specific trials, short follow-ups, and lack of standardized CBT protocols.

This narrative review, conducted with systematic search elements, highlights both the strengths and methodological boundaries of current evidence. The findings emphasize the need for rigorously designed, long-term, and culturally adapted studies, as well as for integrating CBT principles into dental education and interdisciplinary care.

Clinically, early identification of dental anxiety, combined with collaboration between dentists and mental health professionals, can enhance access to effective, psychologically informed care. Emerging tools such as VR-assisted CBT and internet-based delivery models may further increase accessibility and patient engagement.

In conclusion, CBT should be viewed not as an isolated technique, but as the cornerstone of a broader, patient-centered model that integrates psychological science into everyday dental practice—bridging the gap between fear and care.

## 9. Practical and Strategic Recommendations

The findings of this review suggest several actionable directions to strengthen both clinical practice and research in the field of dental phobia:

1. Enhancing Clinical Practice

- Integrate brief anxiety and phobia screening tools into routine dental intake to facilitate early identification of patients at risk of treatment avoidance.
- Provide dental professionals with training in behavioral management techniques, including graded exposure, communication strategies, and relaxation methods.
- Encourage interdisciplinary collaboration between dentists and mental health professionals trained in CBT to ensure continuity of psychological support.

2. Improving Accessibility and Innovation

- Expand the availability of digital and virtual reality-assisted CBT (iCBT, VR-CBT), particularly for children, remote populations, and patients with special healthcare needs.
- Develop culturally adapted CBT models that reflect diverse sociocultural and linguistic contexts, promoting inclusivity and patient engagement.

3. Strengthening Research Quality

- Conduct well-powered RCTs specifically focused on dentophobia, using standardized treatment protocols and validated outcome measures.
- Include long-term follow-up and implementation studies to assess treatment sustainability, feasibility, and cost-effectiveness.
- Apply hybrid effectiveness implementation frameworks to explore the integration of CBT and adjunctive interventions (e.g., VR, EMDR, hypnosis) into standard dental care.

4. Building Policy and Educational Frameworks

- Incorporate psychological and behavioral science principles into dental education curricula to enhance clinicians’ confidence in managing anxiety-prone patients.
- Promote policy and reimbursement structures that support the inclusion of evidence-based psychological services within dental healthcare systems.

## 10. Take-Home Message

CBT remains the most empirically grounded and adaptable intervention for managing dental phobia. Its future success depends on multidisciplinary integration—linking psychology, dentistry, technology, and health policy—to create accessible, sustainable, and patient-centered care models. Bridging these domains will be essential for transforming dental anxiety management from an isolated psychological concern into a routine component of comprehensive oral healthcare.

## Figures and Tables

**Table 1:** Comparative overview of interventions for dental phobia.

Intervention	Target Populations	Evidence Base	Main Outcomes	Limitations
**CBT**	Adults, children, trauma-exposed patients	Multiple RCTs, meta-analyses	Long-term reduction in dental phobia, improved treatment adherence	Requires trained professionals, time-intensive
**VR (Virtual Reality)**	Children, patients with special healthcare needs	RCTs, scoping reviews, [27]	Reduced anxiety/pain during procedures, enhanced engagement	Short-term effects; less effective for complex adult procedures
**iCBT/Digital CBT**	Adolescents, adults, remote populations	RCTs, systematic reviews	Scalable, cost-effective, comparable efficacy to face-to-face CBT	Requires digital access; adherence may vary
**EMDR**	Trauma-related dental fear	Case series, reviews	Reprocessing traumatic dental memories, reduced avoidance	Limited high-quality RCTs; specialized training needed
**Hypnosis**	Adults, selected pediatric cases	Meta-analyses, umbrella reviews	Reduced anxiety, improved cooperation	Variable training standards; skepticism in practice
**Pharmacological approaches (benzodiazepines, nitrous oxide, GA)**	Broad populations, urgent or invasive care	Clinical practice, trials	Immediate compliance during dental procedures	Short-lived; risks of dependency; not curative
**Relaxation/Behavioral distraction**	Children, anxious adults	Small RCTs, observational studies	Reduced anticipatory anxiety, easy to implement	Less durable than CBT; limited long-term benefit

## Data Availability

No new data were created for this literature review.
